# Evaluation of Prognosis in Patients with Lung Adenocarcinoma with Atypical Solid Nodules on Thin-Section CT Images

**DOI:** 10.1148/ryct.220234

**Published:** 2024-01-11

**Authors:** Mengwen Liu, Lin Yang, Xujie Sun, Xin Liang, Cong Li, Qianqian Feng, Meng Li, Li Zhang

**Affiliations:** From the Department of Diagnostic Radiology (M. Liu, Q.F., M. Li, L.Z.), Department of Pathology (L.Y., X.S.), Medical Statistics Office (X.L.), and Medical Records Room (C.L.), National Cancer Center/National Clinical Research Center for Cancer/Cancer Hospital, Chinese Academy of Medical Sciences and Peking Union Medical College, No. 17 Panjiayuan Nanli, Chaoyang District, Beijing 100021, China.

**Keywords:** Adenocarcinoma, Atypical Solid Nodules, CT, Disease-free Survival, Lung, Prognosis, Pulmonary

## Abstract

**Purpose:**

To evaluate the clinicopathologic characteristics and prognosis of patients with clinical stage IA lung adenocarcinoma with atypical solid nodules (ASNs) on thin-section CT images.

**Materials and Methods:**

Data from patients with clinical stage IA lung adenocarcinoma who underwent resection between January 2005 and December 2012 were retrospectively reviewed. According to their manifestations on thin-section CT images, nodules were classified as ASNs, subsolid nodules (SSNs), and typical solid nodules (TSNs). The clinicopathologic characteristics of the ASNs were investigated, and the differences across the three groups were analyzed. The Kaplan-Meier method and multivariable Cox analysis were used to evaluate survival differences among patients with ASNs, SSNs, and TSNs.

**Results:**

Of the 254 patients (median age, 58 years [IQR, 53–66]; 152 women) evaluated, 49 had ASNs, 123 had SSNs, and 82 had TSNs. Compared with patients with SSNs, those with ASNs were more likely to have nonsmall adenocarcinoma (*P* < .001), advanced-stage adenocarcinoma (*P* = .004), nonlepidic growth adenocarcinoma (*P* < .001), and middle- or low-grade differentiation tumors (*P* < .001). Compared with patients with TSNs, those with ASNs were more likely to have no lymph node involvement (*P* = .009) and epidermal growth factor receptor mutation positivity (*P* = .018). Average disease-free survival in patients with ASNs was significantly longer than that in patients with TSNs (*P* < .001) but was not distinguishable from that in patients with SSNs (*P* = .051).

**Conclusion:**

ASNs were associated with better clinical outcomes than TSNs in patients with clinical stage IA lung adenocarcinoma.

**Keywords:** Adenocarcinoma, Atypical Solid Nodules, CT, Disease-free Survival, Lung, Prognosis, Pulmonary

*Supplemental material is available for this article*.

Published under a CC BY 4.0 license.

## Graphical abstract

**Figure:**
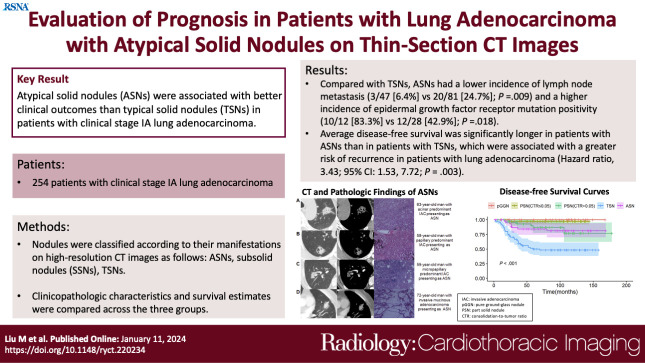

**Summary**

Atypical solid nodules were associated with better clinical outcomes than typical solid nodules in patients with clinical stage IA lung adenocarcinoma.

**Key Points**

■ Compared with typical solid nodules (TSNs), atypical solid nodules (ASNs) had a lower incidence of lymph node metastasis (three of 47 [6.4%] vs 20 of 81 [24.7%]; *P* = .009) and a higher incidence of epidermal growth factor receptor mutation positivity (10 of 12 [83.3%] vs 12 of 28 [42.9%]; *P* = .018).
■ Average disease-free survival was significantly longer in patients with ASNs than in patients with TSNs, and TSNs were associated with greater risk of recurrence in patients with lung adenocarcinoma (hazard ratio: 3.43; 95% CI: 1.53, 7.72; *P* = .003).

## Introduction

Studies have reported age, race, sex, cancer location, pathologic type, airway spread through air space, and TNM staging as prognostic factors for lung cancer (1–5). Nodule features on thin-section CT images, especially nodule consistency that describes the appearance of a lung nodule, have also been proved to be important prognostic factors for lung adenocarcinoma. A ground-glass opacity component in lung adenocarcinoma indicates a favorable prognosis. Even in patients with stage IA1 lung cancer with the highest survival rate, the size of the solid component and ground-glass opacity significantly affect prognosis (6–9). This may be because ground-glass opacity correlates with the lepidic component, and the solid component correlates with the invasive component (6,10,11). Therefore, the eighth edition of the TNM staging system of lung cancer suggested using the size of the invasive component to describe T staging (12).

The Fleischner Society guidelines recommend using contiguous thin CT sections to measure the long and short dimensions of solid components (13). However, not all solid components are typical and easily measured. Studies of part-solid nodules (PSNs) with difficult-to-measure solid components demonstrate that such PSNs are less invasive and rarely have lymphatic invasion (14–16). This suggests that different distributions of solid and other components have prognostic implications. In clinical practice, we have identified a specific type of solid nodule with an atypical solid component, namely multiple pseudocavities, within the solid component. A pseudocavity is defined as "an oval or round area of low attenuation in lung nodules, masses or areas of consolidation" (17).

However, to our knowledge, studies on the atypical solid component in solid nodules have not been reported. In this study, we evaluated the clinicopathologic characteristics and prognosis of lung adenocarcinoma presenting as solid nodules with atypical solid components on thin-section CT images.

## Materials and Methods

This retrospective study was approved by the institutional review board of Cancer Hospital Chinese Academy of Medical Sciences (approval no.: NCCN2017B-026), and the requirement for informed consent was waived.

### Patient Selection

**Figure 1:**
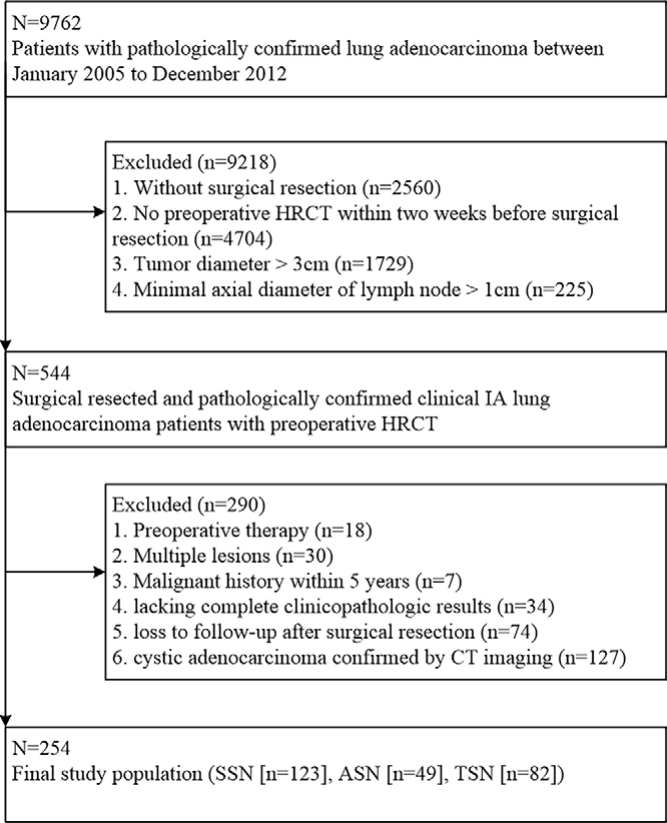
Flowchart shows the patient selection process in the study. ASN = atypical solid nodule, HRCT = high-resolution (thin-section) CT, SSN = subsolid nodule, TSN = typical solid nodule.

Between January 2005 and December 2012, we retrospectively reviewed data from 9762 patients with pathologically confirmed lung adenocarcinoma at our hospital. Patients who underwent surgical resection and thin-section CT less than 2 weeks before surgical resection were included. The exclusion criteria were as follows: average tumor diameter greater than 3 cm or minimal axial diameter of lymph node greater than 1 cm on CT images; no chest CT scans within 2 weeks before surgical resection or CT section thickness greater than 1.25 mm; lung adenocarcinoma associated with cystic airspaces (18); multiple lesions; unavailable pathologic slices or clinicopathological data; preoperative therapy (eg, radiation therapy, chemotherapy, or targeted therapy); previous malignancy with evidence of disease within 5 years; and loss to follow-up since the surgical discharge. Finally, 254 patients were included in this study (Fig 1).

### Evaluation of Clinicopathologic Characteristics

For our analysis, clinical characteristics, including age, sex, smoking history, surgical procedure (sublobar resection and lobectomy), and treatment (surgery alone and adjuvant chemoradiotherapy plus surgery) were evaluated. Tumors were classified in accordance with the 2011 International Association for the Study of Lung Cancer/American Thoracic Society/European Respiratory Society classification and the 2021 World Health Organization classification. We defined adenocarcinoma with a size less than or equal to 1 cm in diameter as small adenocarcinoma and pathologic stage IA adenocarcinoma as early-stage adenocarcinoma (19,20). Tumors were divided into two groups according to histologic subtype: lepidic growth adenocarcinoma (including precursor glandular lesions, minimally invasive adenocarcinoma, and lepidic predominant invasive adenocarcinoma [IAC]) and nonlepidic growth adenocarcinoma (including acinar predominant IAC, papillary predominant IAC, micropapillary predominant IAC, solid predominant with mucin production IAC, variants of predominant IAC, and invasive mucinous adenocarcinoma) (21). Epidermal growth factor receptor *(EGFR)* mutations were detected in tumor tissue and plasma DNA samples from 64 patients using an amplification refractory mutation system or direct DNA sequencing.

### CT Examinations and Interpretation

**Figure 2:**
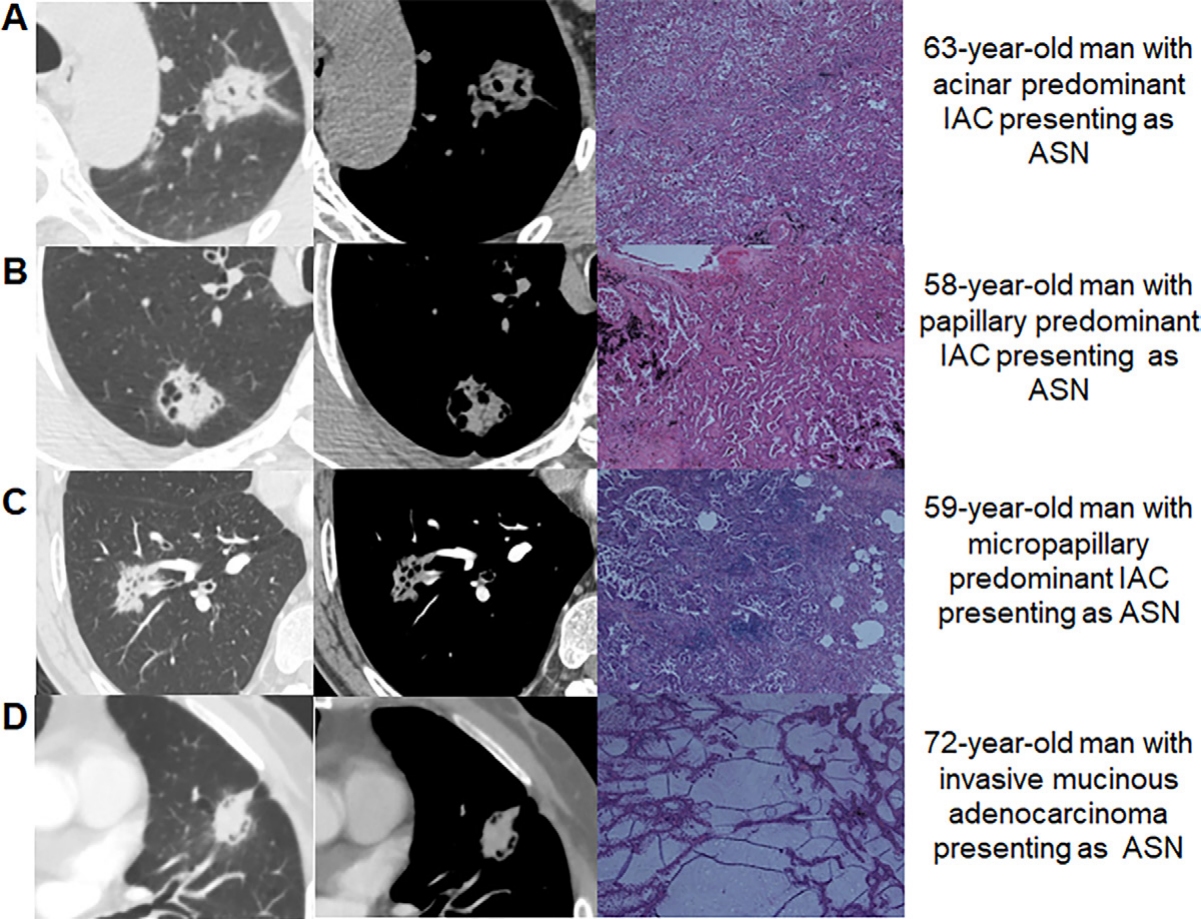
CT and pathologic findings of atypical solid nodules (ASNs). **(A)** Images in a 63-year-old man with acinar predominant invasive adenocarcinoma (IAC) presenting as ASN. The first two CT images depict the ASN, captured in the lung and mediastinal windows, respectively, using nonenhanced CT scans. The third image shows a pathologic specimen of ASN (hematoxylin-eosin stain; magnification, ×40). **(B)** Images in a 58-year-old man with papillary predominant IAC presenting as ASN. The first two CT images depict the ASN, captured in the lung and mediastinal windows, respectively, using nonenhanced CT scans. The third image shows a pathologic specimen of ASN (hematoxylin-eosin stain; magnification, ×40). **(C)** Images in a 58-year-old man with micropapillary predominant IAC presenting as ASN. The first two CT images depict the ASN, captured in the lung and mediastinal windows, respectively, using contrast-enhanced CT scans. The third image shows a pathologic specimen of ASN (hematoxylin-eosin stain; magnification, ×40). **(D)** Images in a 72-year-old man with invasive mucinous adenocarcinoma presenting as ASN. The first two CT images depict the ASN, captured in the lung and mediastinal windows, respectively, using nonenhanced CT scans. The third image shows a pathologic specimen of ASN (hematoxylin-eosin stain; magnification, ×40).

Among the 254 patients, 119 (46.9%) underwent contrast-enhanced scans, 127 (50.0%) underwent nonenhanced scans, and eight (3.1%) underwent both contrast-enhanced and nonenhanced scans. The scans were performed using 16- or 64-section spiral CT scanners (Discovery ST, LightSpeed Ultra, LightSpeed VCT, or ProSpeed AI CT by GE Medical Systems; or Aquilion by Toshiba Medical Systems) at full inspiration. The scanning parameters were tube potential of 120 kVp and auto tube current settings. Reconstruction thicknesses were 1.25 mm or 1.0 mm with 0.8-mm intervals. Two independent thoracic radiologists (L.Z. and M. Li, with 10 years of experience in chest CT) reviewed the preoperative CT images on an Advantage workstation 4.6 (GE HealthCare) while blinded to the clinicopathological results. All thin-section CT images were evaluated on both the lung window (width = 1500 HU; level = -600 HU) and the mediastinal window (window width = 350 HU; level = 40 HU). For quantitative characteristics, including nodule size (the average of the maximal long-axis and perpendicular maximal short-axis measurements in the same plane), solid component size (the maximum diameter), and consolidation-to-tumor ratio (CTR), the average value measured by the two radiologists was adopted in this study. Considering the different biologic behaviors of PSNs with CTR greater than 0.5 and PSNs with CTR less than or equal to 0.5, these two groups of PSNs were analyzed separately (22). Three morphologic features—nodule type (atypical solid nodule [ASN], subsolid nodule [SSN], or typical solid nodules [TSN]), nodule consistency (pure ground-glass nodule [pGGN], PSN, or solid nodule), and invasive lobe—were interpreted. When there was a discrepancy in the interpretation of the morphologic features, a final consensus was reached by group discussion. All measurements followed The Fleischner Society’s recommendations for measuring pulmonary nodules at CT (23). We defined a solid nodule with multiple pseudocavities (≥3) as an ASN, which shows a sievelike appearance in both the lung window and mediastinal window compared with a TSN (Fig 2).

### Postoperative Follow-up

All patients who underwent sublobar resection or lobectomy were followed up from the day after surgery. Postoperative follow-up procedures consisted of physical examination, chest radiography every 3 months, and chest CT scans every 6 months for the first 2 years after surgery. Thereafter, chest radiography was performed every 6 months, and chest CT examination was performed annually. The median follow-up period was 78 months. Survival outcomes and disease progression were obtained through a review of medical records, and telephone interviews were conducted by trained staff members. If a patient or family member could not be reached on the follow-up date, the date and survival information were censored on the date of the last follow-up. Average disease-free survival (DFS) was selected as the end point. DFS was defined as the time from surgery to the date of metastasis.

### Statistical Analysis

The frequency distribution and descriptive statistics were determined for all variables. The data are expressed as the means ± SDs when normally distributed or as the median (IQR) when the normality assumptions were not met. The Kolmogorov-Smirnov test was used to test the normality assumptions. The clinicopathologic differences among SSN, ASN, and TSN were analyzed by using the *t* test and Wilcoxon rank sum test for parametric and nonparametric continuous variables and the χ^2^ test or Fisher exact test for categorical variables. One-way analysis of variance or Kruskal-Wallis sum test was used to analyze the difference in continuous variables among three groups. Average DFS was analyzed using the Kaplan-Meier method, and survival curves were generated. Multivariable Cox regression analyses were performed to determine the prognostic implications of ASNs and other types of nodules, with adjustment for other potential prognostic factors. Statistical analyses were performed by an author (X.L.) using SPSS statistical software version 25.0 (IBM) and R software version 4.1.1 (R Foundation for Statistical Computing). *P* < .05 was considered to indicate a statistically significant difference. Bonferroni correction was adopted for multiple analyses, and the corrected *P* value was calculated based on the number of comparisons.

## Results

### Clinical and Pathologic Findings

The clinical characteristics and the CT and pathologic findings in the 254 patients (median age, 58 years [IQR, 53–66]; 152 women, 102 men) with clinical stage IA lung adenocarcinoma are presented in Table 1 and Table S1. Among the 254 patients, 49 (19.3%) were categorized as having ASNs, 123 (48.4%) as having SSNs, and 82 (32.3%) as having TSNs. Of the 123 patients with SSNs, 34 (27.6%) had pGGNs, 39 (31.7%) had PSNs with CTR less than or equal to 0.5, and 50 (40.7%) had PSNs with CTR greater than 0.5. Six patients did not undergo lymph node dissection. Of the 248 patients who underwent lymph node dissection, 224 patients (90.3%) had no pathologic lymph node involvement, 16 patients (6.5%) had pathologic N1 disease, and eight patients (3.2%) had pathologic N2 disease. Among the 254 total patients, the T stage was upgraded in 112 (44.1%) after surgical resection (107 tumors involved the visceral pleura, one tumor was larger than 3 cm, and four tumors were larger than 3 cm and also involved the visceral pleura). Among the 64 patients who had *EGFR* mutations detected, 26 (40.6%) patients had *EGFR* mutation-negative findings and 38 (59.4%) had *EGFR* mutation-positive findings. Values for entire nodule diameter, solid component size, and CTR of each PSN are shown in Table S2. The clinicopathologic characteristics of the patients with ASNs, pGGNs, PSNs with CTR less than or equal to 0.5, PSNs with CTR greater than 0.5, and TSNs are summarized in Table S3.

**Table 1:**
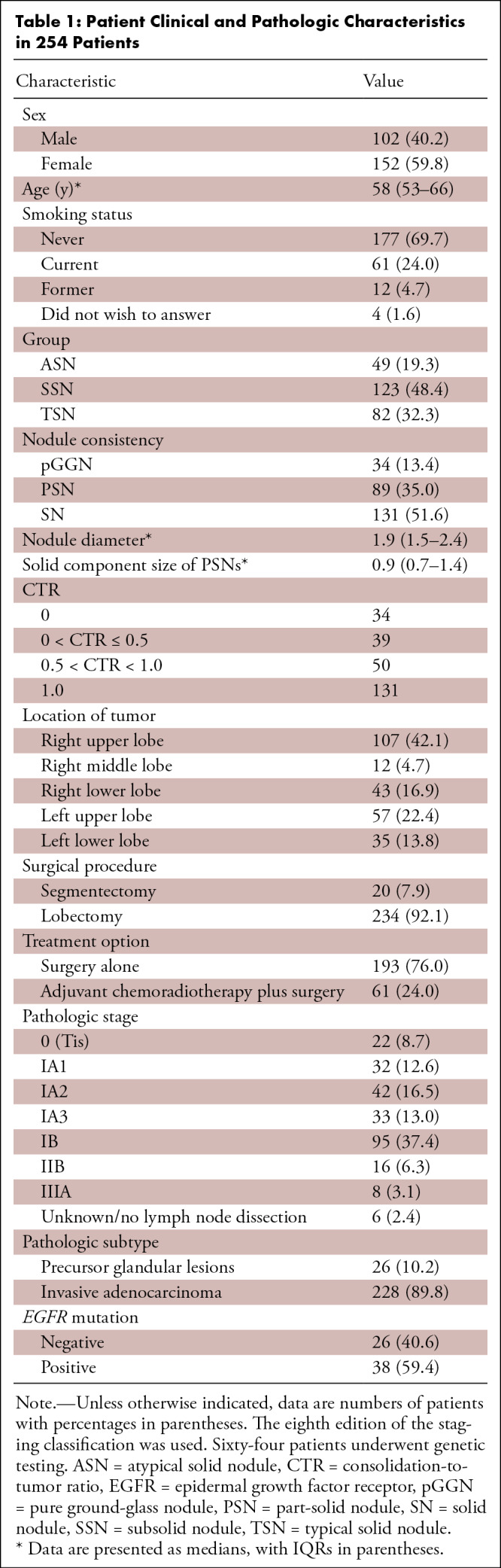
Patient Clinical and Pathologic Characteristics in 254 Patients

### Comparison of ASNs and TSNs

Among the 131 solid nodules, 49 were ASNs and 82 were TSNs. We found no evidence of differences in sex (*P* = .604), age (*P* = .376), smoking status (*P* = .168), small adenocarcinoma (*P* > .999), early-stage adenocarcinoma (*P* = .449), lepidic growth adenocarcinoma (*P* = .332), or tumor differentiation (*P* = .067) between patients with ASNs and TSNs. Compared with the TSN group, patients with ASNs were more likely to have no lymph node involvement (44 of 47 [93.6%] vs 61 of 81 [75.3%]; *P* = .009) and *EGFR* mutation positivity (10 of 12 [83.3%] vs 12 of 28 [42.9%]; *P* = .018) (Table 2).

**Table 2:**
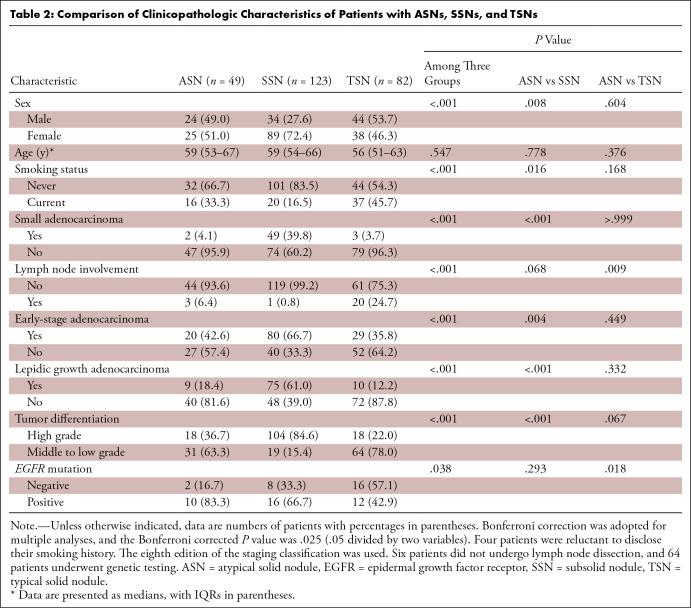
Comparison of Clinicopathologic Characteristics of Patients with ASNs, SSNs, and TSNs

### Comparison of ASNs and SSNs

We found no evidence of differences between the ASN and SSN groups in terms of age (*P* = .778), lymph node involvement (*P* = .068), or *EGFR* mutation status (*P* = .293). Compared with patients with SSNs, patients with ASNs were more likely to smoke (16 of 48 [33.3%] vs 20 of 121 [16.5%]; *P* = .016) and have nonsmall adenocarcinoma (47 of 49 [95.9%] vs 74 of 123 [60.2%]; *P* < .001) and advanced-stage adenocarcinoma (27 of 47 [57.4%] vs 40 of 120 [33.3%]; *P* = .004). The percentage of patients with nonlepidic growth adenocarcinoma was higher among those with ASNs compared with SSNs (40 of 49 [81.6%] vs 48 of 123 [39.0%]; *P* < .001). The percentage of middle- or low-grade differentiated tumors in patients with ASNs was higher than that in patients with SSNs (31 of 49 [63.3%] vs 19 of 123 [15.4%]; *P* < .001) (Table 2).

Compared with patients with PSNs, those with ASNs were more likely to be male (24 of 49 [49.0%] vs 25 of 89 [28.1%]; *P* = .014) and have nonsmall adenocarcinoma (47 of 49 [95.9%] vs 58 of 89 [65.2%]; *P* < .001), nonlepidic growth adenocarcinoma (40 of 49 [81.6%] vs 42 of 89 [47.2%]; *P* < .001), and middle- to low-grade adenocarcinoma (31 of 49 [63.3%] vs 18 of 89 [20.2%]; *P* < .001). There was no evidence of differences in age (*P* = .915), smoking status (*P* = .047), lymph node involvement (*P* = .124), early-stage adenocarcinoma (*P* = .056), or *EGFR* mutation (*P* = .676) between the two groups (Table 3).

**Table 3:**
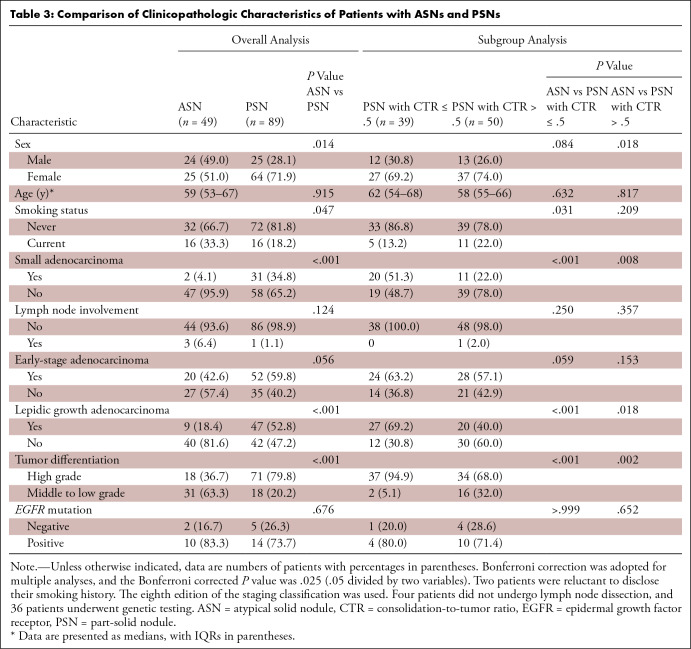
Comparison of Clinicopathologic Characteristics of Patients with ASNs and PSNs

Compared with patients with PSNs with CTR less than or equal to 0.5, those with ASNs were more likely to have small adenocarcinoma (two of 49 [4.1%] vs 20 of 39 [51.3%]; *P* < .001), lepidic growth adenocarcinoma (nine of 49 [18.4%] vs 27 of 39 [69.2%]; *P* < .001), and high-grade tumor differentiation (18 of 49 [36.7%] vs 37 of 39 [94.9%]; *P* < .001). We found no evidence of differences in sex (*P* = .084), age (*P* = .632), smoking status (*P* = .031), lymph node involvement (*P* = .250), early-stage adenocarcinoma (*P* = .059), or *EGFR* mutation (*P* > .999) between each group. Compared with patients with PSNs with CTR greater than 0.5, patients with ASNs were more likely to have small adenocarcinoma (two of 49 [4.1%] vs 11 of 50 [22.0%]; *P* = .008) and high-grade tumor differentiation (18 of 49 [36.7%] vs 34 of 50 [68.0%]; *P* = .002). We found no evidence of differences in sex (*P* = .018), age (*P* = .817), smoking status (*P* = .209), lymph node involvement (*P* = .357), early-stage adenocarcinoma (*P* = .153), lepidic growth adenocarcinoma (*P* = .018), or *EGFR* mutation (*P* = .652) between the two groups (Table 3).

### Survival Analysis

During a median follow-up of 78 months (IQR, 33–113), two patients died of lung adenocarcinoma, and recurrence was observed in 56 (22.0%) of the 254 patients. These included two (4.0%) local-regional relapses and six (12.2%) distant relapses in the 49 patients with ASNs, 0 relapses in the 34 patients with pGGNs, 0 local-regional relapses and one (2.6%) distant relapse in the 39 patients with PSNs with CTR less than or equal to 0.5, 0 local-regional relapses and seven (14.0%) distant relapses in the 50 patients with PSNs with CTR greater than 0.5, and two (2.4%) local-regional relapses and 38 (46.3%) distant relapses in the 82 patients with TSNs.

The recurrence rate among patients with ASNs (16.3% [eight of 49]) was significantly lower than that of patients with TSNs (48.8% [40 of 82]; *P* < .001) but was not significantly different from that of patients with SSNs (6.5% [eight of 123]; *P* = .076) (Table 4). We found no evidence of differences in recurrence rate between patients with ASNs and those with pGGNs (0 of 34), between patients with ASNs and those with PSNs with CTR less than or equal to 0.5 (2.6% [one of 39]), and between patients with ASNs and those with PSNs with CTR greater than 0.5 (14.0% [seven of 50]) by SSN subgroup analysis (all corrected *P* > .017; *P* = .018, *P* = .04, and *P* = .747, respectively) (Table S4). The Kaplan-Meier estimated average DFS rate at 60 months was 81.2% (95% CI: 75.1, 87.3) in patients with ASNs, 94.2% (95% CI: 91.9, 96.5) in patients with SSNs, and 49.9% in patients with TSNs. Average DFS in patients with ASNs was significantly longer than that in patients with TSNs (*P* < .001) but was not distinguishable from that of patients with SSNs (*P* = .051). Subgroup analyses revealed an estimated 60-month average DFS rate of 97.2% (95% CI: 94.5, 99.9) in patients with PSNs with CTR less than or equal to 0.5 and 87.3% (95% CI: 82.0, 92.6) in patients with PSNs with CTR greater than 0.5. The 60-month average DFS rate in patients with pGGNs could not be estimated because no recurrence (0 of 34) was observed in the follow-up period.

**Table 4:**
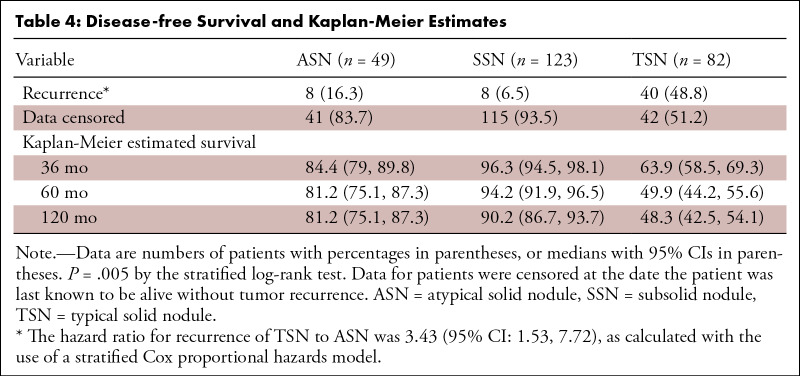
Disease-free Survival and Kaplan-Meier Estimates

**Figure 3:**
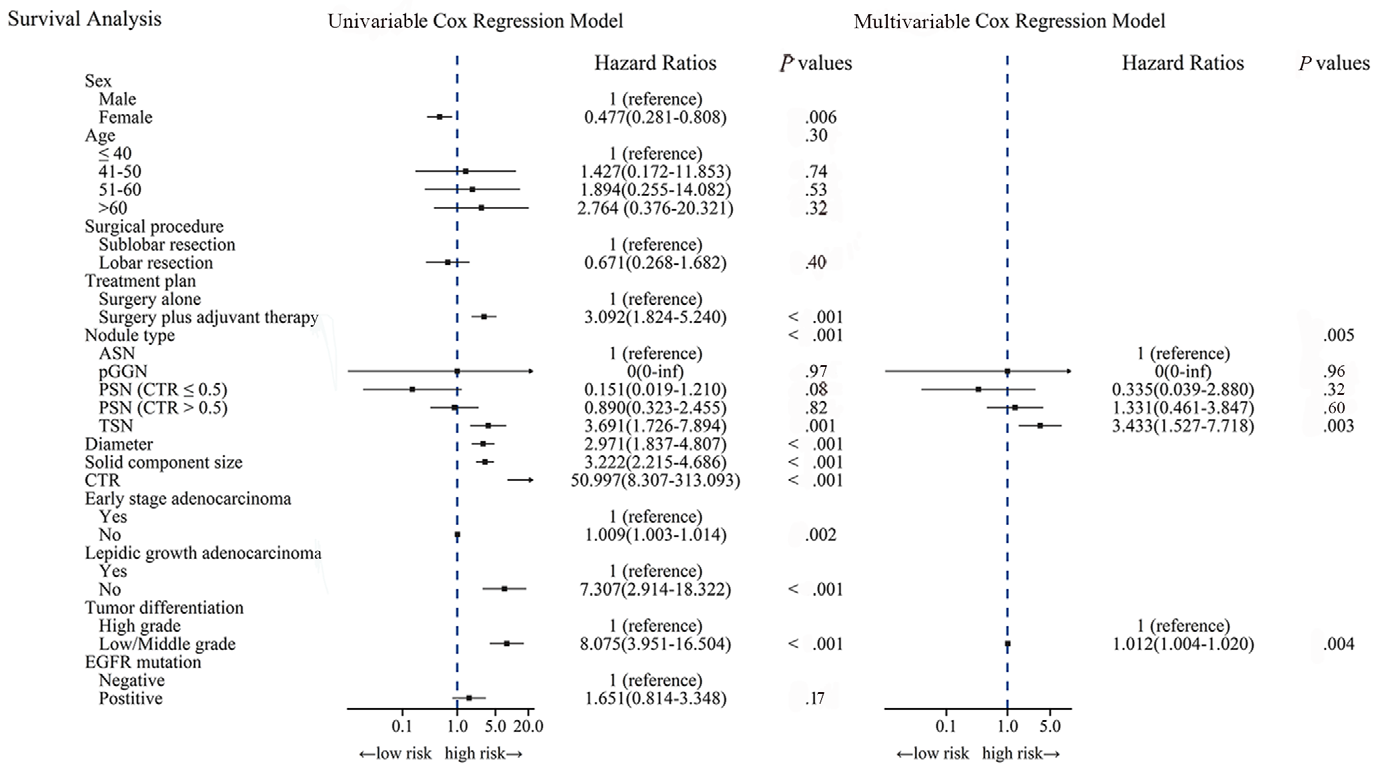
Uni- and multivariable Cox regression analysis of disease-free survival for nodule type. ASN = atypical solid nodule, CTR = consolidation-to-tumor ratio, EGFR = epidermal growth factor receptor, pGGN = pure ground-glass nodule, PSN = part-solid nodule, TSN = typical solid nodule.

**Figure 4:**
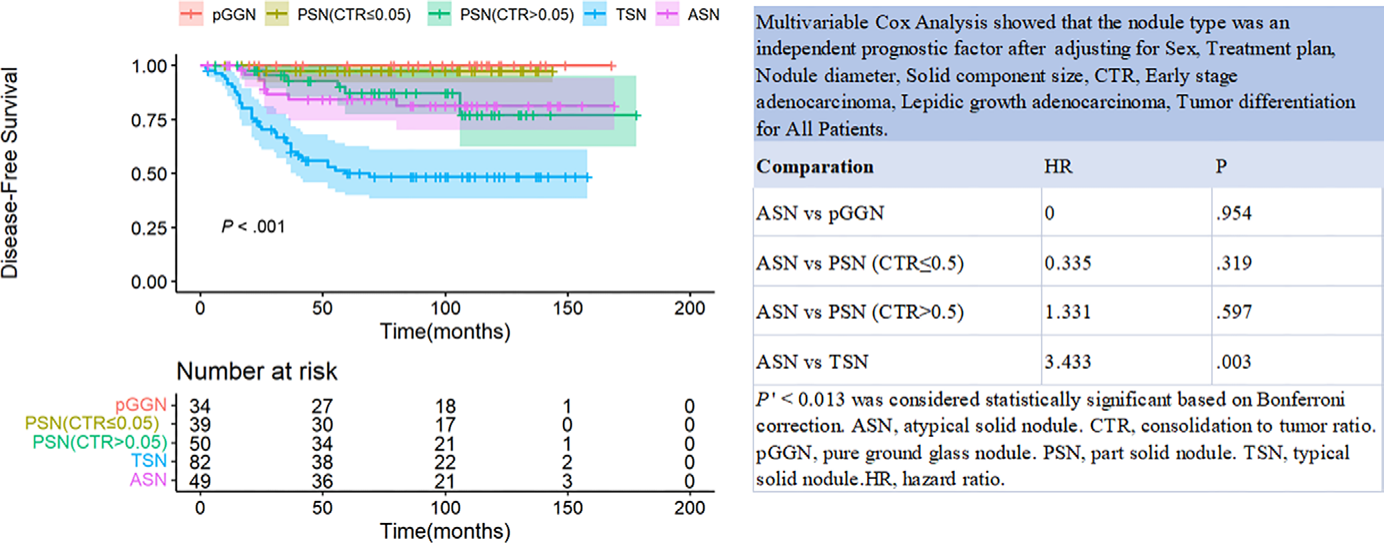
Disease-free survival curves of patients with pure ground-glass nodules (pGGNs), part-solid nodules (PSNs) with consolidation-to-tumor ratio (CTR) less than or equal to 0.5, PSNs with CTR greater than 0.5, atypical solid nodules (ASNs), and typical solid nodules (TSNs). HR = hazard ratio.

The proportional hazards assumption test on the nodule type yielded a *P* value of .60 (Fig S1). After adjusting for sex, treatment plan, nodule diameter, solid component size, CTR, pathologic stage, pathologic subtype, and tumor differentiation in multivariable Cox analysis, nodule type was an independent prognostic factor of average DFS (*P* = .005) (Fig 3). Compared with ASNs, TSNs were associated with greater risk of recurrence in patients with lung adenocarcinoma (hazard ratio: 3.43; 95% CI: 1.53, 7.72; *P* = .003). However, there was no evidence of differences in DFS between patients with ASNs and those with pGGNs, PSNs with CTR less than or equal to 0.5, or PSNs with CTR greater than 0.5 (*P* = .954, *P* = .319, and *P* = .597, respectively) (Fig 4).

## Discussion

In this study, we found that the lymph node metastasis and recurrence rates in patients with ASNs were similar to those in patients with SSNs and significantly lower than those in patients with TSNs. Multivariable Cox regression analysis demonstrated that nodule type was an independent factor for prognosis. The risk of recurrence in patients with TSNs was 3.43 times that of patients with ASNs. Therefore, we believe that ASN may be a special solid nodule with a similar prognosis to SSN, and that ASN should be treated differently from TSN in terms of clinical management.

Lung adenocarcinoma containing ground-glass opacities has a favorable prognosis, possibly because the pathologic pattern of this type of lung adenocarcinoma is lepidic-predominant (10,24–27). Previous research identified lepidic growth as the predominant pathologic subtype of lung adenocarcinoma with pseudocavities (28,29), while acinar and papillary predominant IAC were the most common subtypes in this study (37 of 49 [75.5%]). This inconsistency in findings might be attributed to the fact that previous studies encompassed subsolid and solid nodules, whereas this research focused solely on solid nodules with more than three pseudocavities. Despite the difference in histologic subtypes, both this study and prior investigations have reported a favorable prognosis for adenocarcinomas with pseudocavities.

Although the prognoses of the ASN and SSN groups were similar, the percentage of patients with a lepidic component was lower among those with ASNs (nine of 49 [18.4%] vs 75 of 123 [61.0%]; *P* < .001). Nonetheless, although the predominant pathologic subtypes for both the TSN and ASN groups were nonlepidic adenocarcinomas (*P* = .332), the ASN group demonstrated a more favorable prognosis in comparison to the TSN group. It is difficult to distinguish the two types of solid nodules based on the histologic subtype. Therefore, imaging may be an important means to distinguish ASN and TSN. Recognition of ASNs can provide more information for the selection of treatment plans and the evaluation of prognosis.

Among patients with ASNs, most (44 of 47 [93.6%]) were pathologically identified as having N0 disease, one patient (2.1%) as having N1, and two patients (4.3%) as having N2. These patients with ASNs were less likely to have lymphatic metastasis (44 of 47 [93.6%] vs 61 of 81 [75.3%]; *P* = .009) and were associated with longer average DFS (*P* < .001) compared with patients with TSNs. There was no evidence of a difference between patients with ASNs and those with TSNs in small adenocarcinoma (*P* > .999), early-stage adenocarcinoma (*P* = .449), lepidic growth adenocarcinoma (*P* = .332), or tumor differentiation (*P* = .067), but there was a significant difference in *EGFR* mutation status (*P* = .018). We speculate that the reason may be genetic heterogeneity. Lung adenocarcinoma is a highly heterogeneous tumor that involves various oncogenic genetic alterations, which may impact the prognosis (30). Studies have found that lung cancer with *EGFR* mutations has a lower rate of lymph node metastasis and better prognosis than *EGFR* mutation-negative lung cancer (31,32). In our study, the ASN group was more likely to be *EGFR* mutation-positive (10 of 12 [83.3%] vs 12 of 28 [42.9%]; *P* = .018) and less likely to have lymph node involvement (44 of 47 [93.6%] vs 61 of 81 [75.3%]; *P* = .009) than the TSN group, which is consistent with previous studies. Given that we included patients with a relatively long follow-up period, some surgical specimens were stored for so long that genetic testing could no longer be performed, and we were unable to explore the other genetic differences between ASN and TSN. Therefore, further research is needed to study this issue.

Our study had several limitations. First, the data were derived from a single hospital, and second, the study was retrospective; hence, a multicenter prospective study is needed to confirm our findings. Third, because atypical solid components are difficult to measure, we did not assess the impact of the size of atypical solid components on lung cancer prognosis. Artificial intelligence–based computer-aided measurement systems may be helpful in assessing the impact of size in the future. Fourth, the exclusion of patients with multiple lesions might have introduced selection bias because they represent a subset of the overall patient population. It is essential to acknowledge this limitation and interpret the results with caution, particularly when generalizing the findings to a broader population. Finally, we did not study the pathologic bases of the sievelike structure on thin-section CT images because we found that the regional air spaces were poorly displayed given the lack of attention during the pathologic sampling process. We speculated that the sievelike structures in ASNs might be spared parenchyma, normal or ectatic bronchi, or focal emphysema. We will prospectively compare the pathologic images with thin-section CT images to analyze the pathologic basis of ASNs in further studies.

In conclusion, patients with ASNs had a lower lymphatic metastasis rate and better prognosis than patients with TSNs. Our study suggests that lung adenocarcinoma manifesting as ASNs is a special type of lung cancer that should be treated differently from TSNs in clinical management.
